# The IL-17 family in diseases: from bench to bedside

**DOI:** 10.1038/s41392-023-01620-3

**Published:** 2023-10-11

**Authors:** Longjie Huangfu, Ruiying Li, Yamei Huang, Shan Wang

**Affiliations:** 1 School of Stomatology, Harbin Medical University, Harbin, 150001 P. R. China; 2 Department of Oral Pathology, School of Stomatology, Hainan Medical University, Haikou, 571199 P. R. China; 3 Department of Stomatology, The Second Affiliated Hospital of Hainan Medical University, Haikou, 570216 P. R. China

**Keywords:** Drug development, Immunotherapy, Tumour immunology

## Abstract

The interleukin-17 (IL-17) family comprises six members (IL-17A–17F), and recently, all of its related receptors have been discovered. IL-17 was first discovered approximately 30 years ago. Members of this family have various biological functions, including driving an inflammatory cascade during infections and autoimmune diseases, as well as boosting protective immunity against various pathogens. IL-17 is a highly versatile proinflammatory cytokine necessary for vital processes including host immune defenses, tissue repair, inflammatory disease pathogenesis, and cancer progression. However, how IL-17 performs these functions remains controversial. The multifunctional properties of IL-17 have attracted research interest, and emerging data have gradually improved our understanding of the IL-17 signaling pathway. However, a comprehensive review is required to understand its role in both host defense functions and pathogenesis in the body. This review can aid researchers in better understanding the mechanisms underlying IL-17’s roles in vivo and provide a theoretical basis for future studies aiming to regulate IL-17 expression and function. This review discusses recent progress in understanding the IL-17 signaling pathway and its physiological roles. In addition, we present the mechanism underlying IL-17’s role in various pathologies, particularly, in IL-17-induced systemic lupus erythematosus and IL-17-related tumor cell transformation and metastasis. In addition, we have briefly discussed promising developments in the diagnosis and treatment of autoimmune diseases and tumors.

## Introduction

Interleukin 17 (IL-17) is an important cytokine that has several roles in host defense responses against mucosal infections; additionally, it is a primary pathologic cytokine and therapeutic target in a number of autoimmune, inflammatory illnesses, and cancers. The functions of IL-17 in vivo are not only limited to inflammation, but are also closely associated with both physiological and pathological processes.^1–3^ Various studies have analyzed the molecular mechanisms and pathways underlying IL-17 signaling, providing a theoretical basis for the role of IL-17 in establishing and assisting host defense systems, and inducing tissue regeneration under physiological conditions.^4^ In addition, it can induce autoimmune responses and promote the onset and development of cancer under pathological conditions.^5,6^

**Fig. 1:**
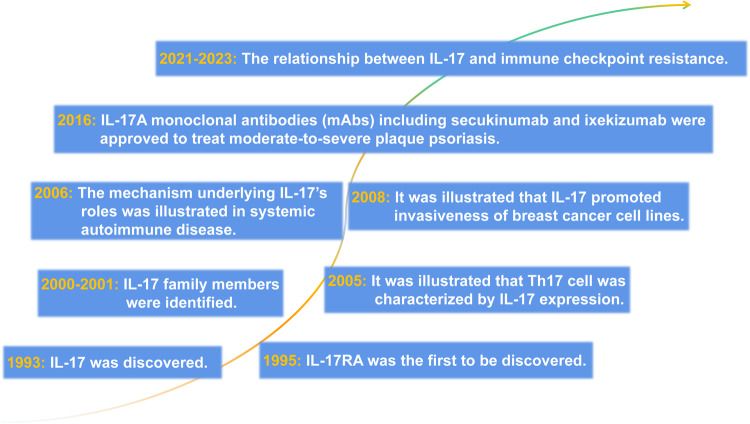
The timeline for IL-17 family research progress

Unless otherwise specified, for our purposes, IL-17 refers to IL-17A, which is a member of the IL-17 family. This family now consists of six structurally-related cytokines: IL-17A, IL-17B, IL-17C, IL-17D, IL-17E (IL-25), and IL-17F.^7^ Of the six members, IL-17A was the first to be identified and is the most significant and studied cytokine in the IL-17 family. It is also formally known as cytotoxic T-lymphocyte-associated antigen 8 (CTLA-8). IL-17A was cloned from T cell hybridomas in 1993^8^ (Fig. 1). Other IL-17 family members were identified through subsequent expression sequence tags and large-scale sequencing of the genomes of several vertebrate species^9–12^ (Fig. 1). Subsequently, human and rat IL-17A proteins were isolated. The human IL-17A monomer is a 155-amino acid glycoprotein. To synthesize the 35 kDa homologous dimer for expression, the 23-amino acid signal peptide in the N-terminus is cleaved, and a disulfide bond is formed.^13^ In the IL-17 family, IL-17F (produced by helper T [Th] cells) is the most similar to IL-17A,^14^ having 55% sequence homology. These ILs can form IL-17F homodimers, IL-17A homodimers, or IL-17A-IL-17F heterodimers that are secreted extracellularly to bind to IL-17 family receptors for signal transduction. However, in 2005, a new subset of CD4^+^ T helper cells (Th17) characterized by IL-17 expression was discovered^15^ (Fig. 1). Although Th17 cells are generally considered to be the primary source of IL-17, natural killer T cells, CD8^+^ T cells, γδ T cells, dendritic cells, macrophages, and other cells also produce this cytokine and are referred to as type 17 cells.^16,17^ Although T-cell receptor (TCR) activation is required for CD4^+^ and CD8^+^ T-cell IL-17 synthesis, innate immune cells primarily produce IL-17 because of the presence of other inflammatory cytokines, particularly IL-1 and IL-23.^18^ Neutrophils may also serve as a source of IL-17 during infection, although this is still debated.^19^

Several studies have focused on understanding the mechanisms of IL-17 transcription, translation, and regulation and have aimed to regulate its expression or block its signaling for the treatment of immunological diseases and tumors related to this cytokine.^20–22^ In 2016, the IL-17A monoclonal antibodies (mAbs) secukinumab and ixekizumab were approved to treat moderate-to-severe plaque psoriasis.^17^ In recent years, the association between IL-17 and tumors has been extensively studied.^23–25^ However, a more comprehensive understanding of the mechanisms of IL-17 signal transduction is required to improve the efficacy of IL-17-related therapies in treating inflammatory and autoimmune diseases and for the precise treatment of tumors.

In this review, we have summarized the molecular mechanisms underlying the IL-17 signaling pathway and its defense and repair functions in the body under physiological conditions. In addition, we have discussed the role of IL-17 in inducing autoimmune responses and inflammatory diseases under pathological conditions and promoting cancer initiation, development, and progression and we present our perspectives and opinions on the treatment of inflammatory and cancerous diseases.

## IL-17 signaling pathways

### Receptors associated with IL-17

**Fig. 2:**
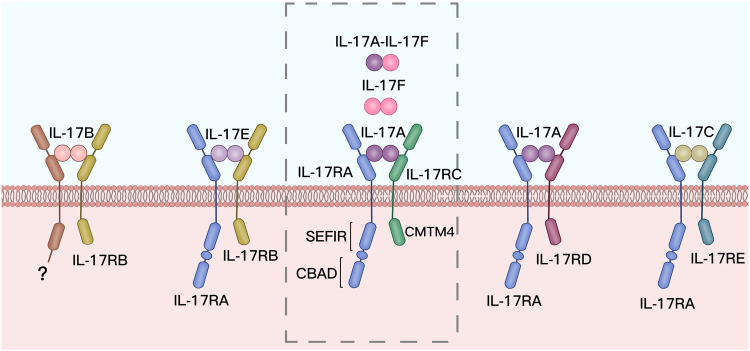
IL-17 cytokine and receptor families. IL-17 family comprises six cytokines. The IL-17 receptor family consists of five distinct receptors that share the SEFIR domain, a cytoplasmic motif. IL-17RA is the common subunit for all the receptors, and the CBAD domain is unique to IL-7RA. Different subunits combine with each other to form different heterodimer receptors. MediBang Paint was used to generate this figure

The IL-17 receptor (IL-17R) family consists of five subunits, namely, IL-17RA, IL-17RB, IL-17RC, IL-17RD, and IL-17RE.^7^ IL-17RA was the first to be discovered (in 1995) (Fig. 1). The IL-17R monomer contains a long intracellular type I transmembrane protein segment, which consists primarily of three domains: fibronectin (FN)1, FN2, and SEFIR. The intracellular segment of IL-17RA also contains a Toll/IL-1 receptor (TIR)-like loop domain and a CCAAT/enhancer-binding protein-beta (C/EBPβ)-activation domain (CBAD).^26^ Different receptor subunits are assembled into heterodimers on cell membrane surfaces to bind ligands for signal transduction^27^ (Fig. 2). IL-17A and IL-17F activate downstream signaling pathways for signal transduction, primarily through IL-17RA and IL-17RC heterodimers. IL-17A/F also activates a downstream signaling pathway through this receptor.^28^ Three similar IL-17 dimers differ in their binding ability to IL-17R—IL-17A/A > IL-17A/F > IL-17F/F.^29^ Although IL-17R is widely distributed in the body, its receptors are not uniformly distributed. For example, IL-17RA is widely expressed, but IL-17RC is primarily expressed in non-hematopoietic epithelial cells and mesenchymal cells, which restricts IL-17A signaling to these two cell types.^28,30^ A recent study revealed that IL-17RD may be a subunit of IL-17A, but it does not bind to IL-17F.^29,31^ IL-17RD instead binds to IL-17RA to form a heterodimeric receptor that can only bind to IL-17A/A.^32^ Therefore, IL-17-related signaling pathways can also be activated in cells expressing IL-17RA and IL-17RD. Due to the distinct receptors and ligands of the IL-17 family of signaling molecules, and the unique recognition specificity of the alternative subunits, various IL-17 members can play diverse roles in different parts of the body. The distribution of these receptors throughout the body and their ligand-binding properties are still under investigation.

IL-17A signaling begins when IL-17R specifically binds to the SEFIR isotype present in Act1 through its intracellular SEFIR domain.^33,34^ Act1 is also functionally associated to the connection to IκB kinase and stress-activated protein kinases (CIKS). It is a multifunctional IL-17R adapter and the primary modulator in the IL-17 signaling pathway to activate downstream signaling molecules. Almost all IL-17-mediated signaling pathways require Act1.^35–37^ Upon binding to IL-17R, Act1 recruits various tumor necrosis factor (TNF) receptor-associated factors (TRAFs) via its TRAF-binding sequence to initiate a separate downstream pathway.^38,39^ In addition, Act1 has E3 ligase activity, and can bind directly to RNA, providing the necessary conditions for IL-17-induced transcription and post-transcriptional activation of target genes.^40,41^ Thus, blocking IL-17R-specific binding to Act1 effectively attenuates IL-17-mediated diseases.^42^ To avoid continuous Act1 activity in vivo, Act1 is catalyzed by the F-box E3 ubiquitin ligase and β-transducing repeat protein (β-TrCP) after sufficient activation of its downstream signaling pathways, and it is then modified and degraded by the lysine (K) 48-linked ubiquitin chain.^43^

The distal domain in the cytoplasmic tail of IL-17RA is distinct from SEFIR. This domain is required to activate the transcription factor C/EBP-β and is therefore termed CBAD.^42–45^ CBAD is associated with TRAF3 and the ubiquitin-editing enzyme A20.^46,47^ The latter two molecules inhibit signal transduction, such that the cytoplasmic tail domain of IL-17RA negatively regulates signal transduction.

Moreover, IL-17RC is required for cell responses to IL-17A–17F signaling, and interactions between these two related cytokines. Thus, altering IL-17RC levels on cell surfaces may be useful for treating IL-17-mediated diseases. CKLF-like MARVEL transmembrane domain-containing protein 4 (CMTM4) was identified by Knizkova as a novel component of IL-17RSC,^48^ which in conjunction with IL-17RC and IL-17RA, forms the ligand-engaged IL-17RSC and is necessary for IL-17-induced cellular responses.

### Transcriptional and post-transcriptional signaling regulated by IL-17

The IL-17-mediated signaling pathways have several similarities and differences with the Toll-like receptor (TLR)/IL-1R signaling pathway. Although the two are homologous, Act1 functions differently in these two pathways. Act1 is an unused adapter in the TLR/IL-1R family but is an essential multifunctional adapter in the IL-17 pathway.^35^ Specifically, Act1 binds to IL-17R to activate its own E3 ligase activity, and TRAF6 binds to the TRAF-binding motif in Act1. This causes conjugation of a K63-linked polyubiquitin chain with TRAF6.^40^ Ubiquitinated TRAF6 activates the expression of inflammatory transcription factor-induced genes through the nuclear factor kappa-light-chain-enhancer of activated B cells (NF-κB) pathway and contributes to the activation of three mitogen-activated protein kinase (MAPK) pathways—the c-Jun N-terminal kinase (JNK), p38, and extracellular signal-regulated kinase (ERK) pathways.^17^

**Fig. 3:**
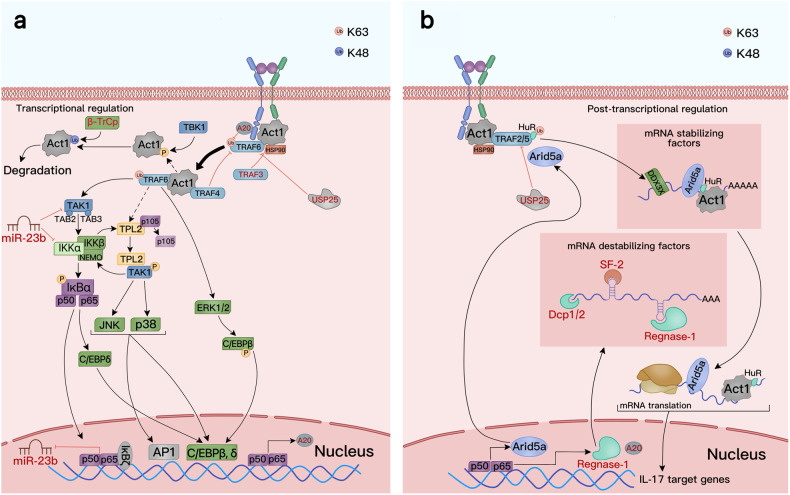
Common IL-17 signaling pathways. **a** Transcriptional regulation. IL-17A binds to its heterodimer receptor, and Act1 binds to TRAF6 to activate the NF-κB and MAPK pathways, promoting the occurrence and progression of inflammation. IL17A can induce various feedback regulatory responses by inducing deubiquitinase (A20, USP25). TBK and β-TrCp mediate Act1 degradation. TRAF3 and TRAF4 inhibit Act1 degradation by competing with TRAF6. IκBζ inhibits the function of miR-23b and promotes inflammation. Hsp90 maintains Act1 integrity at the protein level.^3^ **b** Post-transcriptional regulation. IL-17 binds to its receptor via the Act1-TRAF2-TRAF5 complex to activate downstream signaling pathways mediating mRNA stabilization and translation of IL-17 target genes. DDX3X, ARID-5A, Act1, and HuR are ribosomal binding proteins (RBPs) that increase mRNA stability. Other RBPs, including SF-2, Dcp1/2, and Regnase-1, accelerate mRNA decay. ARID-5A, HuR, and Act1 are also involved in the translation of IL-I7 target genes. IL-17 signaling-induced ARID-5A and Regnase-1 play different roles in modulating the expression of target genes. USP25 can also inhibit the formation of the Act1-TRAF2-TRAF5 complex by inhibiting downstream signaling pathways.^3^ MediBang Paint was used to generate this figure

TRAF6 ubiquitination provides a scaffold for the recruitment and activation of transforming growth factor β-activated kinase (TAK) 1. Activation of the TAK1 complex is necessary for the recruitment and activation of the NF-κB kinase inhibitor (IKK) complex composed of IKKα, IKKβ, and IKKγ/NEMO subunits.^35,36,49^ IKK then phosphorylates the NF-κB subunits, and the labeled IκB is hydrolyzed by protease enzymes to expose NF-κB’s nuclear localization signal, which is released and binds to target inflammatory genes to promote their transcription^50^ (Fig. 3a). In the IL-17-mediated NF-κB pathway, the expression of many target genes is regulated by NF-κB inhibitors (IκBζ), which drives transcription. IκBζ levels are increased by IL-17 via transcriptional and translational induction; IL-17 further induces the mRNA and protein syntheses of numerous target genes in cooperation with NF-κB,^51^ and inhibits miR-23b (an IL-17 signaling inhibitor) to promote the expression of target genes.^51–54^

The significance of IL-17’s role in the MAPK pathway varies according to cell background.^55^ When stimulated by IL-17, the activated IKK complex mediates the phosphorylation of the NF-κB subunit p105, releases tumor progression locus 2 (TPL2) from p105, and activates p38 and JNK via the TPL2-TAK1 axis. In addition, the TPL2-TAK1 axis can activate IKK via positive feedback to further enhance target gene induction. IL-17-induced experimental autoimmune encephalomyelitis (EAE) was attenuated in mice with impaired p38-MAPK signaling, EAE was aggravated by increased IL-17 activity in the appellative experimental condition in mice lacking the p38-MAPK inhibitor MKP1 (Dusp1).^56^ These are reflective of the significance of IL-17 in the MAPK pathway.

Both the IkBζ and C/EBP transcription factors produced in the IL-17-mediated signaling pathway are additional regulators of IL-17 signaling, and have redundant binding sites on the IL-17 promoter.^57^ Inflammatory response can be enhanced when necessary by increasing the expression of IkBζ and C/EBP.^58^ An inflammatory response that is too strong and prolonged will damage healthy tissues. Therefore, the TRAF6-mediated IL-17 signaling pathway is regulated by several mechanisms to prevent damage caused by excessive inflammatory responses. A20 (also known as tumor necrosis factor alpha-induced protein 3, *Tnfaip3*), which is upregulated by IL-17A via NF-κB, binds to the CBAD of IL-17RA and removes polyubiquitin chains on TRAF6, thereby inhibiting IL-17 signaling through negative feedback regulation.^59,60^ Similarly, ubiquitin-specific peptidase 25 (USP25) can deubiquitinate TRAF6, thereby blocking the IL-17 signaling pathway.^61^ In addition, TRAF4 can compete with TRAF6 to bind the TRAF-binding motif of Act1. Similarly, TRAF3 binds to the CBAD of IL-17RA and competes with TRAF6 to suppress IL-17-induced inflammatory responses. Thus, the absence of either TRAF3 or TRAF4 increases the expression of IL-17-dependent target genes, resulting in an enhanced IL-17-mediated inflammatory response.^46,62^

Induction of IκBζ by IL-17 in fibroblastic reticular cells induces the production of carnitine palmitoyltransferase 1A (CPT1A), which is a requisite for the increased glucose absorption necessary for cell survival.^63,64^ Notably, an itaconate derivative (a metabolite from the tricarboxylic acid cycle) inhibits IL-17-mediated activation of IκBζ and its downstream target genes in keratinocytes.^65,66^ Itaconate-mediated control of IκBζ is an impressive example of how a common metabolic pathway can have immunoregulatory potential.^65,66^

In the previous section, we discussed how the IL-17 pathway promotes the expression of target genes by activating the NF-κB and MAPK pathways, and can promote or inhibit both pathways through feedback regulation (Fig. 3a). However, further studies are required to reveal how these two distinct pathways can be manipulated and modulated in vitro to sustain immune responses and cure diseases.^67^

In addition to enhancing the inflammatory response by increasing target mRNA transcription, the IL-17 pathway promotes the functional activity of numerous target genes by controlling the stability of the transcribed mRNA. The target mRNA produced by IL-17 through the NF-κB and MAPK pathways is inherently unstable due to its 3’ untranslated region (UTR) sequence, which serves as a binding platform for RNA-binding proteins (RBPs). Moreover, IL-17 weakly stimulates the above pathways and can only moderately induce the transcription of inflammatory genes.^17^ Therefore, there is a close correlation between the stability and degradation of the generated inflammatory mRNA and the intensity of inflammation. Common RBPs that enhance mRNA stability include HuR, Act1, AT-rich–interactive domain-containing protein 5A (ARID-5A), and DEAD-box helicase 3 X-linked (DDX3X).^41,67–70^ Other RBPs such as multifunctional RBP splicing factor 2 (SF-2) and ribonuclease regnase-1 can accelerate RNA decay^69–71^ (Fig. 3b).

**Fig. 4:**
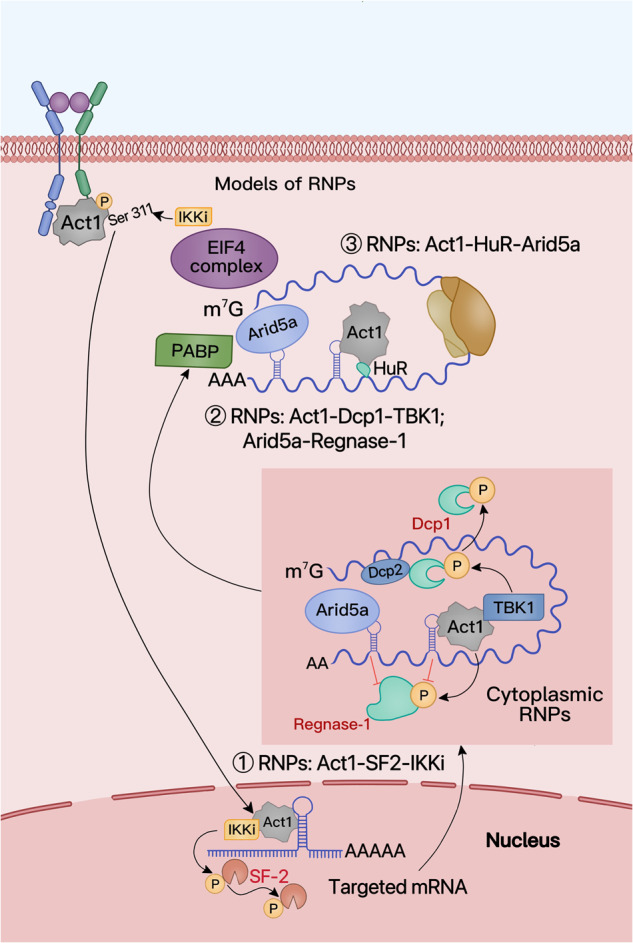
Ribonucleoprotein (RNP) model. The IL-17 signaling pathway regulates mRNA metabolism at different stages through the formation of multiple RNPs. Stimulated by IL-17, IKKi undergoes nuclear transfer after inducing Act1 phosphorylation at Ser311, and binds to the target mRNA through Act1, forming RNP1 to inhibit SF2-mediated target mRNA decay. Act1 follows the mRNA to cytoplasmic granules and Act1-TBK1 syngotes bind to mRNA to form RNP2. The degradation of target mRNA is inhibited by TBK1-mediated phosphorylation of Dcp1/2 (decapping). In addition, Act1-TBK1 and ARID-5A can counteract the effect of Regnase-1 on cytoplasmic RNPs. Finally, Act1 binds to mRNA and promotes the binding of HuR to mRNA to form RNP3, which promotes protein translation. ARID-5A can also promote target gene translation.^3^ MediBang Paint was used to generate this figure

Several mechanisms of IL-17-mediated regulation of mRNA stability via RNPs have been proposed. When the Act1 adapter of IL-17R acts as an RBP, it promotes Act1 phosphorylation at Ser311 by the induction of the IκB kinase I (IKKi), promoting the nuclear translocation of ACT1-IKKi and its binding to the specific stem ring structure of the target gene through its SEFIR domain. This leads to SF-2 phosphorylation, thereby reducing SF2-mediated target mRNA decay.^69^ Act1 phosphorylation also interferes with the binding of TRAF6 to Act1 and inhibits the activation of NF-κB.^72^ In cytoplasmic ribonucleoproteins, Act1 inhibits the activity of mRNA-release enzymes decapping protein-1 (Dcp1) and -2 (Dcp2) through TANK binding kinase 1 (TBK1)-mediated phosphorylation, thereby increasing mRNA stability.^41^ Regnase-1-mediated degradation of mRNA is inhibited by IL-17-TBK1 phosphorylation.^73^ In addition, Act1 promotes the binding of HuR to mRNA, enabling the target gene to undergo simultaneous multi-ribosome translation.^41,70^ In addition to the above three pathways, IL-17 can induce the expression of ARID-5A to increase the expression of target genes or inhibit their degradation. ARID-5A competes with Regnase-1 for 3’ UTR binding sites to stabilize the target mRNA and promote its translation, thus enhancing target gene expression^50,56^ (Fig. 4).

Inhibitory mechanisms that act on mRNA at the post-transcriptional level function to prevent damage from excessive inflammatory reactions. Regnase-1 is a potent inhibitor of the IL-17 signaling pathway. Its usual mode of action is to bind and degrade mRNA undergoing translation.^74^ As mentioned above, ARID-5A can compete with regnase-1 for the 3’ UTR binding site of target genes to inhibit their degradation by regnase-1. Whether ARID-5A or Regnase-1 prevails in this scenario depends on their respective levels in the body. IL-17 can induce an increase in cellular ARID-5A and Regnase-1 levels through the NF-κB pathway.^75^ Therefore, maintaining the balance between ARID-5A and Regnase-1 is a dynamic process that can inhibit inflammation when the expression of Regnase-1 is increased (Fig. 3b). It can also restrict Regnase-1’s function in promoting target gene expression. IL-17 induces the production of multiple mRNAs; however, the effects of RBPs on different mRNAs vary. Thus, it may be possible to develop specific targeted drugs that block various RBPs based on their mechanisms of action to treat diseases. For example, chlorpromazine can reduce IL-17-induced inflammation by specifically targeting ARID-5A.^50^ In addition to acting on RBPs, the direct binding of drugs to target mRNA can be attempted using targeting mechanisms that promote or inhibit the degradation of target genes by competing with RBPs. In preclinical autoimmunity models, drugs can alleviate autoimmune diseases by specifically binding to the 3’ UTR of Chemokine ligand 1 (Cxcl1, also termed KC) to block Act1 binding.^41^

Post-transcriptional mechanisms may also affect IL-17’s involvement in T cell differentiation. MicroRNA (miR)-133b and miR-206 were the first miRNAs discovered to be co-regulated with IL-17 in T cells, despite having no functional impact on cytokine production.^76^ However, other data have shown that during thymocyte development, the miR-181a/b-1 cluster is extensively expressed and positively controls the potency of TCR signaling.^77,78^ Prinz et al. investigated the role of miR-181a/b-1 in γδ T cells and observed that miR-181a/b-1 deficiency did not affect thymic γδ T cell numbers or differentiation towards IL-17- or interferon-gamma (IFNγ)-producing effectors.^79^

### IL-17-regulated synergistic signaling interactions

Besides its direct effects on the expression of genes associated with IL-17R-induced inflammation, IL-17 cooperates with other ligands to participate in numerous signaling pathways. IL-17 interacts with IFNγ, IL-13, transforming growth factor-beta (TGF-β), and other cytokines to induce inflammation. In addition, it interacts with other non-inflammatory cytokines, such as epidermal growth factor receptor (EGFR), fibroblast growth factor 2 (FGF2), caspase recruitment domain family member 14 (CARD14), and NOTCH to influence tissue repair, autoimmune diseases, and tumor development.^80^ Several signaling molecules interact synergistically with IL-17. Because more is known about FGF2 and CARD14 in relation to IL-17, these two are described in detail below.

**Fig. 5:**
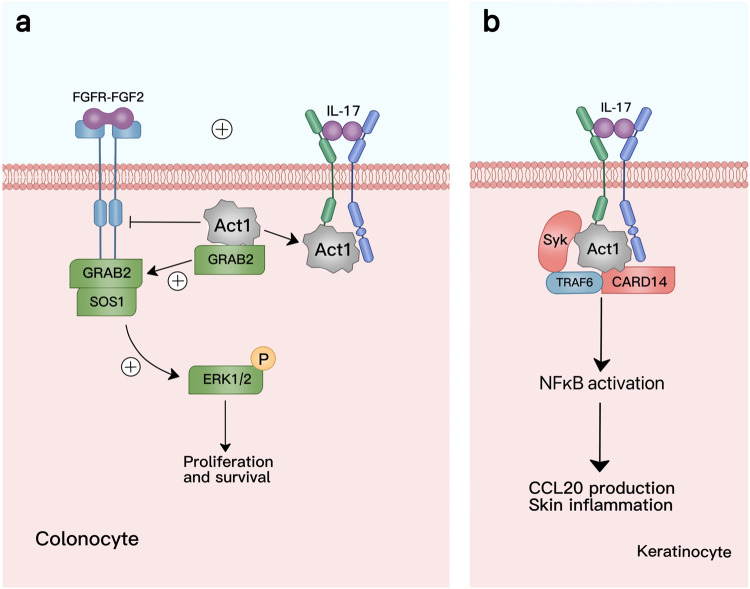
Synergistic signaling regulated by IL-17. **a** IL-17A synergizes the fibroblast growth factor (FGF) signaling pathway. Act1 binds directly to GRAB2, the FGF receptor (FGFR) in colonic epithelial cells, and inhibits the activation of the FGF2 signaling pathway. Stimulated by IL-17A, Act1 is recruited by IL-17R and separates from GRAB2. Free GRAB2 is recruited to FGFR, binds to Son of sevenless homolog 1 (SOS1), induces downstream phosphorylation of extracellular-regulated kinase 1/2 (ERK1/2), and promotes epithelial cell proliferation and survival. **b** Integration of IL-17 signaling pathway in autoimmune diseases. In keratinocytes, under the action of IL-17, Act1 and TRAF6 can form complexes with GARD14 and SyK to activate the NF-κB pathway and promote the production of CCL20 and skin inflammation. MediBang Paint was used to generate this figure

IL-17 can interact with FGF2 in the colonic epithelium to regulate cell-growth factor signaling pathways.^80^ In intestinal cells, Act1 binds to growth factor receptor-bound protein 2 (GRAB2), an adapter of fibroblast growth factor receptors (FGFRs). The bound form of the ACT1-Grab2 complex inhibits GRAB2 from binding to the guanine nucleotide exchange factor SOS Ras/Rac guanine nucleotide exchange factor 1 (SOS1), thereby inhibiting the activation of the FGF2 pathway. When IL-17 binds to the receptor, Act1 is separated from GRAB2 via recruitment by IL-17RA, resulting in an increased FGFR recruitment to GRAB2 and enhanced FGF2-induced ERK1/2 phosphorylation (Fig. 5a). Specifically, when IL-17A-induced signaling is enhanced, the proliferative activity of intestinal cells increases, promoting tissue regeneration and tumor development.^81–83^ Therefore, ERK1/2 activation in the intestinal epithelium depends on the combined action of FGF2 and IL-17A.^81^

IL-17’s synergistic activity with other signaling molecules has also been associated with various autoimmune diseases.^84,85^ Drugs targeting IL-17 can be used to effectively treat psoriasis.^86,87^ Researchers are investigating whether antibodies against IL-17 can be used to treat other autoimmune diseases.^88^ CARD14, which is overexpressed in psoriasis, can interact with Act1 and TRAF6 to form a complex to activate the NF-κB pathway, promote the production of C-C motif chemokine ligand 20 (CCL20), and enhance IL-17A-mediated skin inflammation.^89^ In another study, the IL-17A-induced NF-κB pathway was found to be influenced by Syk kinase levels^90^ (Fig. 5b). Therefore, antibodies against IL-17 may be an effective treatment option for psoriasis and other autoimmune diseases.^91–93^ Further research on autoimmune diseases and the identification of additional immune disease-associated cytokines may provide new insights for future treatments.

## IL-17-driven physiological responses

IL-17, a potent pro-inflammatory cytokine, plays a pivotal role in inflammatory processes.^67,94–96^ IL-17 can indirectly induce neutrophil differentiation by stimulating the production of granulocyte colony-stimulating factor (G-CSF), monocyte chemoattractant protein-1 (MCP-1), and CXC chemokines by non-hematopoietic target cells. At the same time, IL-17 deficiency can cause a decrease in neutrophils.^6^ Although IL-17 is an important inflammatory mediator, its unique positive and negative feedback regulatory mechanisms of the signaling pathwaya render it a moderate signaling activator compared to other inflammatory stimuli.

IL-17 is also closely associated with host defense responses. IL-17 can combat bacterial invasion by promoting tertiary immunity in lymphoid tissues. For example, it promotes bronchus-associated lymphoid tissue (iBALT) immunity against intracellular pathogens such as *Mycobacterium tuberculosis*.^97,98^ Moreover, some researchers have suggested that IL-17 is associated with viral immunity.^99,100^ It is also effective against fungi such as *Candida albicans*; previous studies showed that almost all individuals demonstrated a Th17 response against *C. albicans*.^101^ Experimental observations of patients with mutations in the gene encoding IL-17R have suggested widespread prevalence of *C. albicans* and *Staphylococcus aureus* in human mucosal skin infections.^102^ Experiments with mice administered IL-17-blocking antibodies revealed that a certain level of IL-17 effectively prevented candidiasis.^103,104^ IL-17 also boosts immunity against other fungi. In experimental fungal vaccines in mice, IL-17 is required to induce tertiary immunity; for example, by targeting Blastomyces, Histoplasma, and *Coccidioides*.^105–108^

### Involvement of IL-17 in immune responses to various infections

#### Fungal infections

IL-17 polymorphism studies in humans and knockout mouse assays have provided compelling evidence that IL-17-producing cells are necessary for protection against the fungi *Candida* and other infectious pathogens. Individuals with autosomal recessive IL17RA deficiency or ACT1 mutations are predisposed to develop chronic mucocutaneous candidiasis (CMC).^109,110^ Furthermore, dominant-negative mutations are linked to CMC. Signal transducer and activator of transcription 3 (STAT3), a transcription factor involved in the IL-6, IL-21, and IL-23 signaling pathways, is essential for the Th17 cell production. In addition, individuals using anti-IL-17 mAbs are more likely to develop oropharyngeal, esophageal, and cutaneous candidiasis.^111^ Mice deficient in IL-17 or its associated receptors showed an increased fungal load following a fungal challenge in mouse models. Increased renal infection rates and decreased survival were observed in Il17ra-/- animals after systemic *C. albicans* exposure, and were associated with decreased neutrophil recruitment to the kidneys.^112^ Il23p19/ and Il17ra/ mice had more severe oral candidiasis than wild-type mice, whereas Il12p35/ animals did not. These findings indicate that Th17 cells, rather than TH1 cells, are necessary for neutrophil recruitment and defensin production.^113^ Humans and animals lacking IL-17RA are more susceptible to oropharyngeal candidiasis (OPC), exhibit lower CXC chemokine levels, and show decreased recruitment of neutrophils to the oral mucous membranes. The fact that IL-17RA- or ACT1-deficient animals are more vulnerable to OPC than IL-17A-/- mice suggests that IL-17F (as well as IL-17A) contributes to oropharynx antifungal immunity.^104^ The most common cause of fungal infection in humans is *C. albicans*. IL-17 is essential for defense against superficial fungal infections, but its involvement in invasive illness is less clear. *C. albicans* secretes Lip2 lipase, which promotes invasive disease by suppressing the lipid-mediated IL-17 response.^114^

#### Bacterial infections

IL-17 has complex roles in bacterial infections. In vitro experiments demonstrated that IL-17 increases IL-8 release from gastric epithelial cells, which promotes neutrophil chemotaxis.^115^ IL-17 levels are elevated in the gastric mucosa of individuals with a *Helicobacter pylori* infection.^115^ After intranasal challenge with *Klebsiella pneumoniae*, Il17ra/ mice rapidly succumbed to fatal infections.^116^ The authors reported that infection with *K. pneumoniae* resulted in faulty neutrophil recruitment as well as reduced G-CSF and CXCL2 levels in Il17ra/ mice, and were the first to establish a link between IL-17 signaling and neutrophil recruitment.^116^

IL-17 can directly stimulate neutrophil- and macrophage-mediated bacterial killing and indirectly attract neutrophils by increasing chemokine production. The IL-17 response prevents colonization of the nasopharynx by *Streptococcus pneumoniae* via neutrophil recruitment and pneumococcal eradication.^117–119^ However, IL-17 can also increase vulnerability to pulmonary *Acinetobacter baumannii* infection in mice by inhibiting neutrophil phagocytosis during the early stages of infection.^120^

By targeting SIGLEC-F^+^ neutrophils with high NETosis activity and stimulating AMP synthesis, IL-17 plays a crucial role in the resolution of primary and secondary *Bordetella pertussis* nasal mucosal infections in mice.^121^ The role of IL-17 in preventing *Francisella tularensis* infection are indirectly mediated through promoting interferon (IFN) production, which improves macrophage-mediated bacterial killing;^122^ however, this likely reflects the flexibility of Th17 cells, leading to a shift towards IFN production. There are additional data suggesting that IL-17 and IFN collaborate to increase macrophage nitric oxide generation, strengthening defenses against chlamydia infections.^123^ In order to mitigate various host cell processes, *Coxiella* survives in a phagolysosome-like vacuole and uses the Dot/Icm type IVB secretion system (T4BSS) to release bacterial effector proteins into the cytoplasm of the host cell. *Coxiella* T4BSS prevents IL-17-mediated oxidative stress and inhibits IL-17-induced activation of the NF-kB and MAPK pathways. This describes a novel mechanism by which intracellular bacteria circumvent the immune response during the early stages of infection.^124^ IL-17 and IFNγ enhance the abilities of macrophages and neutrophils to kill intracellular *B. pertussis*.^125,126^ Moreover, mouse immunization studies of a potential *M. tuberculosis* vaccine revealed that lung-accumulating IL-17-secreting T cells encourage the generation of chemokines that attract Th1 cells to regulate infection.^97^ These results potentially reflect Th17 cell plasticity in vivo, and they also suggest a collaborative effect of IL-17 on the IFN response to various bacterial infections.

#### Viral infections

Infections caused by viruses such as the influenza virus,^127^ human immunodeficiency virus-1 (HIV-1),^128^ and hepatitis C virus (HCV)^129^ induce antigen-specific Th17 cells. However, the mechanism through which IL-17 affects viral immunity remains unclear. Studies show that IL-17 participates in IL-23-regulated resistance to vaccinia virus infection, but is unlikely to play a dominant role in this response.^130^ Studies on simian immunodeficiency virus (SIV)-infected rhesus macaques indicate that increased systemic spread of *Salmonella typhimurium* from the gut was seen in IL-17 receptor-deficient animals, indicating that IL-17 deficiency results in abnormalities in the mucosal barrier function.^131^

Memory CD4^+^α4^+^β7^hi^T cells that generate IL-17 are preferentially depleted during acute SIV infection. Their loss accelerates the disease course and skews to a Th1-type response.^132^ During a progressive HIV infection, Th17 cells are diminished and Treg cells increase in proportion, resulting in compromised immunological function.^133^ Th17 cells may also contribute to vaccine-induced antiviral immunity by increasing Th1-type memory T cell counts in the female vaginal tract.^134^

IL-17 contributes to effective antiviral immune responses during infection with West Nile virus, adenovirus, and vaccinia virus. However, IL-17 can also promote and exacerbate viral-induced effects during infection with Theiler’s murine encephalomyelitis virus, Coxsackievirus, dengue virus, hepatitis B virus, HCV, and gamma herpesvirus. In addition, IL-17 can have both protective and pathogenic effects during infections with influenza virus, herpes simplex virus, respiratory syncytial virus, SIV, and HIV.^135^

In severe coronavirus disease (COVID-19) cases (caused by severe acute respiratory syndrome coronavirus 2 [SARS-CoV-2]), IL-17 may cause lung inflammation and acute respiratory distress syndrome. Th17 cell populations are increased and activated in patients with COVID-19 that exhibit pulmonary symptoms.^136^ Furthermore, hyperinflammation and lung injury in such patients are correlated with increased Th17 cell responses,^137^ neutrophilia, and increased NETosis.^138^ In addition, IL-17 stimulation is partly driven by *Candida* colonization, and blunted type I/III IFN signaling is a common feature of severe COVID-19 infection.^139^

#### Parasitic infections

By encouraging monocyte and/or macrophage activation, IL-17 has been shown to play protective roles in immunity against several parasites, mainly intracellular protozoa. Neutrophil recruitment is the main mechanism by which IL-17 promotes infection-induced immunopathology in this setting; however, it also plays a minor role in mediating immunity against large multicellular parasites.^140,141^ In mice, IL-17 protects against the protozoan parasite—*Trypanosoma cruzi*—by attracting neutrophils that secrete the immunosuppressive cytokine IL-10, thereby reducing the generation of IFNs and inflammatory responses that can cause liver damage.^142^

Polymorphisms in the IL17A gene or abnormally high expression have been associated with the development of persistent cardiomyopathy after *T. cruzi* infection.^109,143^ In BALB/c mice, IL-17 and IL-22 (produced in response to a DNA vaccine that encodes ROP13) have been linked to defense against *Toxoplasma gondii*.^144^ Anti-IL-17A antibodies that inhibit IL-17 activity reduced collagen deposition and inflammatory cell infiltration in infected C57BL/6 mice livers. Anti-IL-17A monoclonal antibody blocking increased the serum levels of soluble egg antigen (IL)-specific IgGs, indicating that IL-17 typically suppresses this humoral response. These results imply that T cells are the main producers of IL-17, and that IL-17 has a role in the granulomatous, inflammatory, and fibrosing reactions in the livers of *S. japonicum*-infected C57BL/6 mice.^141^

### IL-17-mediated regulation in barrier tissue

In addition to establishing host defense responses, IL-17 plays an important role in barrier maintenance. In normal intestinal epithelial tissue, IL-17 protects the mucosal barrier and controls epithelial infections by maintaining tight junctions between epithelial cells and upregulating antibacterial proteins such as β-defensin and calcitonin (S100A8/9).^5^ When the tissue is damaged, IL-17 can activate and stimulate the function of stem cells at the damaged site, repair damaged tissue, and restore mucosal barriers.^83^ Moreover, IL-17 is essential for wound recovery. The IL-17-induced antimicrobial peptide RegIIIγ can promote skin wound closure,^145^ and tissue plasminogen activator can promote intestinal tissue repair.^146^ Therefore, using anti-IL-17 biologic drugs for disease treatment should be strictly controlled to ensure that the remaining IL-17 in the body is capable of maintaining the integrity of the skin and mucous membranes.^80^

IL-17 is a powerful promoter of barrier tissue healing^147^ and other metabolically demanding processes.^148^ Findings in patients with inflammatory bowel illness, whose symptoms worsened after clinical trials with anti-IL-17A/IL-17RA biologics for Crohn’s disease, were used to deduce the regenerative role of IL-17 in the gut.^149,150^ These findings agree with those from the earlier research in mice, which show that Act1, a crucial IL-17R adapter, stimulates tissue repair in response to gut damage.^35^ This is supported by the finding that deficits in Il17a or IL-17 neutralize the intestinal damage brought on by dextran sodium sulfate in mice.^151–153^ Similar to IL-17’s supportive role in skin wound healing, decreased IL-17 expression in murine skin injury models causes delayed wound closure, weakened barrier integrity, and dysbiosis of the skin’s microbiome.^154,155^ Instead of promoting pathogenic inflammation, such as that seen in psoriasis, IL-17 promotes skin and gastrointestinal tissue repair in homeostatic and acute models of inflammation. IL-17 is required for certain aspects of wound healing, which can be explained by several mechanisms. IL-17 stimulates keratinocytes and epithelial cells to proliferate through mitogenic signaling like the ERK pathway.^45,83,156^ Furthermore, IL-17 promotes the expression of antimicrobial peptides such as β-defensins, RegIII, S100A8/9,^153,154,157,158^ and Steap4 metalloreductase,^156^ which promote proliferation.^153,154,157,158^ IL-17 may control the cellular localization of the tight junction protein—occludin, which has been postulated to underlie gut epithelial barrier function; however, the expression of genes linked to epithelial tight junction integrity were not affected in IL-17A-deficient mice.^153^ Furthermore, it appears that other cytokines present in the local inflammatory environment provide synergistic signals and modulate IL-17-driven tissue healing. Specifically, IL-17 and FGF2 synergistically induce genes that repair damaged epithelia.^80^ In theory, nutrient availability might control IL-17-induced repair responses. However, we currently know very little about how various bioenergetic processes (including glycolysis and FAO) affect IL-17-mediated tissue repair and regeneration.

Cytokines co-regulated with IL-17, such as IL-22, are also important in epithelial cell homeostasis and epithelial cell repair/regeneration after inflammatory damage. In one study, IL-22-depleted mice displayed decreased allergic inflammation and proinflammatory cytokine expression, indicating that IL-22 expression has a protective/restorative effect on tissues after a certain period.^159^ IL-22 has also been demonstrated to activate autophagy in alveolar epithelial cells while IL-17 suppresses it.^160,161^ Autophagy is a crucial mechanism for maintaining epithelial cell homeostasis during cellular stress. In response to respiratory syncytial virus infection, epithelial cells from autophagy-deficient animals generated significant amounts of IL-1 and displayed accelerated IL-17-driven lung disease.^162^ The IL-17/IL-22 axis regulates homeostatic mechanisms, such as autophagy, and is critical in determining adverse outcomes to both viral and noninfectious inflammatory stimuli. Given the pleiotropic nature of IL-17, it is not surprising that, in addition to these beneficial effects, it can contribute to various inflammatory and fibrotic lung disorders.

### IL-17 promotes bone tissue homeostasis and regeneration

In general, increases in IL-17A following bone injuries is believed to enhance bone regeneration, with mouse IL-17A-/-models demonstrating worse bone regeneration.^163^ The JAK2/signal transducer and activator of transcription 3 (STAT3) signaling pathway activates osteoblasts, resulting in osteogenesis and fracture repair.^164^ In psoriatic arthritis (PsA) trials, IL-17A inhibition resulted in significant decreases in minor joint erosion, which was related to inflammatory suppression.^165,166^ Ankylosing spondylitis (AS), like PsA, causes higher levels of IL-17A in patients’ serum and synovial fluid.^164,167^ Emerging evidence suggests that IL-17F plays a significant role in spondyloarthritis spectrum illness and psoriasis, and dual inhibition of IL-17A and IL-17 may be beneficial, particularly in the skin.^168^ IL-17F production in response to inflammatory stimuli can activate the C/EBP protein, which mediates osteoblastogenesis during early fracture repair.^169^ This is reinforced by the high IL-17F levels observed at the fracture callus in a mouse tibial fracture model 3 days after fracture, indicating a role in fracture repair.^170^ There is growing evidence that IL-17A and IL-17F work in concert, and dual inhibition of both cytokines may suppress osteogenesis more effectively.

## IL-17 involvement in pathologic responses

Although transient and controlled IL-17 expression induces physiological reactions for host immune defense mechanisms^171^ and tissue healing,^172,173^ chronic IL-17 activation promotes autoimmunity and cancer by orchestrating harmful responses.^174^

### IL-17 in autoimmunity and inflammation

As a potent proinflammatory cytokine, IL-17 plays an essential role in the inflammatory process and is constantly regulated by the host health status.^38,94–96^ The production and levels of IL-17 maintained in the body are relatively low and stable under normal physiological conditions. In healthy individuals, Th17 cell activation is inhibited by TGF-β, which prevents excessive IL-17 secretion and maintains immune tolerance.^171^ In contrast, Th17 cell activation is enhanced during pathogen invasion, and IL-17 secretion is increased under the combined effects of TGF-β and IL-6, promoting inflammation.^16,175^ The disruption of IL-17 production can lead to autoimmune diseases and tissue destruction. Excessive levels of IL-17 in the body are associated with the development and exacerbation of several autoimmune diseases.^70,71^

#### Skin inflammation

The roles of IL-17 in psoriasis, PsA, and AS are well-established.^176–178^ CMTM4 is a subunit of the IL-17 receptor and mediates psoriasis.^48^ In mice and humans, Th17 cells are found in the dermis of psoriatic skin lesions,^179^ and regulate skin inflammation in response to self-lipid antigens presented by cluster of differentiation 1a (CD1a).^180^ IL-17 activity connects psoriasis and metabolic-associated fatty liver disease.^181^

IL-17E (also termed IL-25), which is abundant in the lesional skin of patients with psoriasis, was found to be mediated by IL-17 in a murine imiquimod-induced psoriasis model. IL-17E injection caused skin inflammation, whereas IL-17E deletions in the germline or keratinocytes increased resistance to imiquimod-induced psoriasis. IL-17E promoted keratinocyte proliferation and the production of inflammatory cytokines and chemokines by activating the STAT3 transcription factor.^182^ The findings show that IL-17E mediates a keratinocyte autoregulatory circuit triggered by IL-17 signaling, which may be targeted when treating patients with psoriasis.^182^

In addition, tissue-resident CD8^+^ T cells that produce IL-17 have been observed in the synovial fluid of patients with PsA.^183^ Other cellular sources of IL-17, such as T cells, neutrophils, and mast cells, as well as IL-23-driven IL-22 induction, may contribute to the pathophysiology of these illnesses.^184^

Numerous potent therapeutics targeting the IL-23/IL-17 pathway are used in clinical settings. Empirical trials have demonstrated that IL-17 family mAbs are beneficial for treating moderate to severe psoriasis (Table 1). Patients with psoriasis experience increased skin clearance after bimekizumab administration, which blocks both IL-17A and IL-17F, in contrast to secukinumab, which only blocks IL-17A.^185^ AS and PsA can be effectively treated with drugs targeting the IL-23-IL-17 pathway.^186^ However, long-term side effects are associated with IL-17 inhibitors, including mucosal and opportunistic infections, which are the most common immune-related adverse events in these patients.^187^

**Table 1:** IL-17 pathway-targeted therapies in autoimmunity and inflammation

mAb to IL-17 pathway in clinal trials/human use	FDA-approved for use date	Targeted therapy marker sites	Indications at the time of FDA approval	Indications	Application number
ustekinumab	09/25/2009	p40 subunit of IL-12 and IL-23	Treatment of patients 12 years older with chronic moderate to severe plaque psoriasis who are candidates for phototherapy or systemic therapy	Psoriasis Crohn’s disease Ankylosing spondylitis Rheumatoid arthritis Psoriatic arthritis Multiple sclerosis GvHD Atopic dermatitis	125261
	09/23/2016		For the treatment of adult patients with moderately to severely active Crohn’s disease (CD) who have failed or were intolerant to treatment with immunomodulators or corticosteroids, but never failed treatment with a tumor necrosis factor (TNF) blocker, or failed or were intolerant to treatment with one or more TNF blockers.		761044
secukinumab	01/21/2015	IL-17A	Treatment of moderate to severe plaque psoriasis in adult patients who are candidates for systemic therapy or phototherapy and who did not respond well to medication applied directly to the skin.	Psoriasis Rheumatoid arthritis Ankylosing spondylitis Psoriatic arthritis Asthma Multiple sclerosis Type 1 Diabetes Crohn’s disease	125504
ixekizumab	03/22/2016	IL-17A	Treatment of moderate to severe plaque psoriasis in adult patients who are candidates for systemic therapy or phototherapy	Psoriasis Rheumatoid arthritis	125521
bimekizumab	N/A	IL-17A and IL-17F	N/A	N/A	N/A
brodalumab	02/15/2017	IL-17RA	Treatment of moderate to severe plaque psoriasis in adults who may benefit from systemic treatment (such as injections or pills) or phototherapy (ultraviolet light treatment) and who did not respond or lost response to other systemic treatments.	Psoriasis Psoriatic arthritis Asthma Crohn’s disease	761032
guselkumab	07/13/2017	IL-23p19	Treatment of adult patients with moderate-to-severe plaque psoriasis who are candidates for systemic therapy or phototherapy.	Psoriasis Rheumatoid arthritis	761061
tildrakizumab	03/20/2018	IL-23p19	Treatment of adults with moderate-to-severe plaque psoriasis who are candidates for systemic therapy or phototherapy.	Psoriasis	761067
risankizumab	04/23/2019	IL-23	Treatment of moderate to severe plaque psoriasis in adults who may benefit from taking injections or pills (systemic therapy) or phototherapy (treatment using ultraviolet or UV light).	Psoriasis	761105
	06/16/2022	IL-23	Treatment of moderate-to-severe plaque psoriasis in adults who are candidates for systemic therapy or phototherapy, active psoriatic arthritis in adults and moderately to severely active Crohn’s disease in adults	Psoriasis	761262

Hidradenitis suppurativa is a chronic, inflammatory, debilitating skin disease that affects hair follicles and is characterized by significant skin infiltration of CD161- and IL-17-expressing Th17 cells.^188^ Secukinumab has moderate efficacy in patients with hidradenitis suppurativa based on open-label pilot clinical trials, and phase III trials are currently being conducted.^189^ Two open-label trials of brodalumab, an IL-17 receptor antagonist, have shown that it may be effective in patients who have not benefited from multiple biologics. However, the results of our real-world efficacy trial fell short of the efficacy achieved in these open-label trials.^190^ IL-17-blocking medications are also used off-label for the treatment of other skin conditions, such as lichen planus or pityriasis rubra pilaris, although secukinumab is ineffective in treating alopecia areata.^191^

#### Inflammatory bowel disease

IL-17 levels are dramatically enhanced in the blood and inflamed mucosa of individuals with active ulcerative colitis or Crohn’s disease.^149,150^ In addition, genome-wide association studies have revealed that a non-synonymous SNP in IL23R is linked to Crohn’s disease.^192^ IL-17 (generated by Th17 cells and/or ILCs and activated by IL-1 and IL-23) has been linked to chronic intestinal inflammation in mouse models of colitis.^193,194^ IL-23 promotes IFNγ production, which works in tandem with IL-17 to mediate intestinal inflammation.^195^ This pathological process may also be mediated by ex-Th17 cells, which are Th17 cells that have transformed to IFNγ-producing cells.^196^ In contrast, in patients with inflammatory bowel disease, clinical studies with IL-17 inhibitors increased the risk of *C. albicans* infections in the intestinal mucosa.^149,150^ However, although IL-17 and Th17 cells can produce inflammation and damage to the gut mucosa, IL-17 and IL-22 perform protective functions against fungal and bacterial gut infections.^197^

Treatment with IL-17 inhibitors is linked to new-onset and exacerbations of inflammatory bowel disease and colitis.^198^ Studies in rat models showed that blocking IL-17 exacerbates inflammation, whereas blocking IL-12p40 or IL-23p19 is protective.^199^ IL-17 and IL-22 play roles in maintaining the barrier integrity of the intestinal epithelium; mice with missing gastrointestinal epithelial cells with functioning ACT1 lose the protective effects of IL-17.^200^ Conversely, inhibiting IL-17F, but not IL-17A, induces protective Treg cells by altering the microbiota,^153^ suggesting that IL-17F may aggravate murine colitis. To study the reported occurrence and features of all unfavorable gastroenterological incidents in IL-17 inhibitor-treated individuals, Caron et al. performed a literature search (up to March 2021). They included 160 clinical randomized trials involving 40,053 individuals. Inflammatory bowel illness was present in 0.4% of individuals treated with IL-17 inhibitors. Other gastrointestinal adverse events with high frequencies included diarrhea, nausea or vomiting, and gastroenteritis. Sixty-one uncontrolled or retrospective studies comprising 16,791 participants were also included. There were 60 (0.36%) cases of inflammatory bowel illness, and 0.6% of patients experienced additional gastrointestinal side effects. For treating psoriasis, PsA, and AS, IL-17 inhibitors are risk-free and efficient. Anti-IL-17 medications have a low frequency of causing new-onset inflammatory bowel disease or aggravating preexisting inflammatory bowel disease.^201^

#### Systemic lupus erythematosus (SLE)

Several studies have shown that IL-17 levels are altered in patients with SLE and that the levels of IL-17 produced in the plasma and target organs are positively linked with disease severity.^202,203^ Interestingly, in two animal models of lupus, decreased IL-17 expression effectively alleviated the pathological changes in SLE.^204^ However, the correlation between IL-17 levels and SLE severity remains unclear. Although it has been observed at the macro-level that IL-17 levels change with changes in symptoms in patients or mice with SLE, subsequent studies have failed to establish a direct relationship.^205,206^ A subsequent meta-analysis confirmed that IL-17 levels were positively correlated with SLE progression. However, the low level of correlation has led researchers to question the intrinsic relationship between IL-17 and SLE.^207^

Various immune cells produce IL-17; however, Th17 cells are the primary producers. In addition to producing IL-17, Th17 cells can produce a variety of proinflammatory cytokines such as IL-21, IL-22, and TNF-α.^208,209^ To determine the true “culprit” underlying pathological changes in SLE, researchers investigated other cytokines associated with Th17 cells. Serum levels of IL-21 and IL-22 have also been found to increase with symptom severity in patients with SLE.^210^ IL-17 can modulate the pathological processes of SLE in humans through the combined action of other cytokines associated with IL-17-producing cells.^210^

Based on the above discussion, we found that Th cells play a role in the pathological changes in SLE by secreting IL-17 and other immune-related cytokines. Inhibiting Th17 cell differentiation can alleviate SLE exacerbations. Several studies have shown that IL-23 is an important promoter of Th17 cell differentiation and factor in Th17 cell development, expansion, and proliferation.^211^ Using anti-IL-23 mAbs for the treatment of active SLE is a promising approach.^30^ Th17 differentiation in vivo is also influenced by the balance between Th17 and Treg cells.^212–214^ In patients with SLE, Treg cells decrease and Th17 cells increase. This Th17/Treg imbalance promotes lupus severity.^215^ Increasing the differentiation of Treg cells in vivo and restoring the Th17/Treg balance using different methods may be another effective way to treat SLE. In addition to the above two pathways, some common factors influence Th17 cell differentiation. For example, activated B cells can stimulate the release of IL-23 from dendritic cells, thus promoting the production of IL-17. Activated monocytes can induce γδ T and mast cells to release IL-17 or activate Th17 cells.^216^ Plasmacytoid dendritic cells activate Th17 cells to secrete IL-17 by secreting large amounts of IFNγ antigen-presenting cells.^216–220^ In addition, Ma et al. showed that upon stimulation with IL-17A (IL-17), a significant subset of IL-17 receptor-expressing plasma cells produced strong anti-double-strand DNA IgG in such patients and murine lupus models. According to reports, IL-17 dramatically improves plasma cell survival through p38-mediated stabilization of the *Bcl-xL* gene^221^ and sustains plasma cells in SLE.^222^ It is possible that blockade of these pathways, either separately or in combination, may be effective for the treatment of SLE.

These collective findings indicate that IL-17 is involved in the occurrence and development of SLE in multiple ways. In addition to relieving lupus by directly targeting IL-17, novel therapies aim to regulate the differentiation and developmental pathways of IL-17-secreting Th17 cells, thereby inhibiting IL-17 secretion and indirectly treating SLE.

#### Other autoimmune and inflammatory diseases

Research using a mouse model of experimental autoimmune uveitis revealed that IL-17 plays a significant role in its pathology,^223^ although both Th1 and Th17 cells have been shown to contribute to this disease.^224^ Secukinumab was not efficacious in clinical trials in treating patients with noninfectious uveitis.^225^ Th17 cells also play a detrimental role in autoimmune diabetes, although therapy with recombinant IL-25 or anti-IL-17 mAbs (which block Th17 cells) are effective in ameliorating the disease.^226^ Moreover, patients with type 1 diabetes have more number of Th17 cells in their blood, and human islet cells respond more aggressively to inflammation when exposed to IL-17.^227^

Despite much uncertainty over the specific function of IL-17 in EAE and multiple sclerosis (MS) and the difficulties in extrapolating the results from mouse research to humans, cumulative data have demonstrated persuasive evidence that IL-17 is a vital pathologic cytokine in EAE and a promising therapeutic target in MS. MS patients’ cerebral fluid contains immune cells that generate IL-17 mRNA.^228^ Moreover, in patients with MS, Th17 cells penetrate the blood-brain barrier and assemble in regions with active lesions.^229^ In a proof-of-concept study, administering anti-IL-17 (secukinumab) therapy to patients with relapsing-remitting MS led to a 67% decrease in the number of new lesions.^230^ Unfortunately, despite promising results in patients with MS and compelling evidence from the EAE animal model, this has not been pursued in extensive clinical trials.

Rheumatoid arthritis (RA) is another disease in which IL-17 has been targeted for therapy, but was ultimately unsuccessful. Patients with RA have high levels of IL-17 in the synovial fluid, which encourages osteoclastogenesis.^231^ Moreover, synovial fibroblasts secrete IL-6, IL-8, and matrix metalloproteinases in patients with RA.^232^ Studies involving mouse models of RA have revealed that Th17 and γδT17 cells mediate autoimmune arthritis,^233,234^ and inhibiting IL-17 reduced joint inflammation and cartilage destruction.^235^ However, clinical trials using IL-17- or IL-23/IL-12p40-targeting antibodies in patients with RA demonstrated little or no efficacy.^236,237^ Uncertain disease heterogeneity or the fact that ex-Th17 cells (which produce IFNγ, but not IL-17), rather than traditional Th17 cells, are elevated in the synovial fluid of individuals with RA may explain the limited therapeutic efficacy of IL-17-targeted drugs in RA.^228,238^ Biopsied synovial tissue samples during the course of RA may be able to shed light on the severity of synovitis in terms of stromal cell proliferation and enrichment of the B cell lineage, and can help evaluate this activity across the disease stages in combination with multiparametric activity scores^199^ to create patient-specific individual taxonomies.^239^

Psoriasis is a chronic, inflammatory, immune-mediated illness that mostly affects the skin. According to emerging data, patients with psoriasis have a disturbed intestinal barrier and frequently suffer from gastrointestinal comorbidities. Furthermore, there is rising evidence of paradoxical cutaneous and intestinal reactions following treatment with biologics in such patients. These reactions highlight the link among the gut-skin axis, microbiome, psoriasis treatment, and the occurrence of paradoxical reactions in psoriasis patients with psoriasis, such as inflammatory bowel disease. Better understanding of how these systems interact can lead to tailored therapies and a decreased incidence of pharmaceutical side effects, increasing patients’ quality of life.^240^

#### IL-17‑targeted therapy for autoimmunity

Several autoimmune diseases (i.e., plaque psoriasis, PsA, obligatory spondylitis, and autoimmune nephropathy) are closely associated with imbalances in IL-17 levels, suggesting that anti-IL-17 antibody therapy could be an effective treatment strategy for psoriasis and AS.^91–93^ Given the essential role of anti-IL-17 therapy in autoimmune diseases, researchers have targeted IL-17 for targeted therapy and achieved significant efficacy.^241–246^ Similar to SLE, antibody therapies against IL-17 utilize the following two main mechanisms: direct targeting of the IL-17 pathway to inhibit inflammation and (or) indirectly inhibiting the IL-17-mediated inflammatory response by regulating Th17 cell differentiation.^244^ Given their roles in autoimmunity, the mAbs—secukinumab (AIN457), ixekizumab (LY2439821), and brodalumab (AMG827), which target the IL-17 signaling pathway, were approved by the United States Food and Drug Administration (FDA) in 2015, 2016, and 2017, respectively, for the treatment of moderate-to-severe psoriasis.^72,73^ These mAbs demonstrated promising efficacy.

##### Secukinumab

Secukinumab (AIN457, Cosentyx), a recombinant human IgG1/kappa mAb, targets IL-17A and prevents it from binding to and interacting with its receptor (IL-17R). The binding prevents the downstream production of proinflammatory cytokines and chemokines that contribute to the onset of various diseases.^247^ Secukinumab was approved by the FDA on January 21, 2015 for the treatment of moderate-to-severe plaque psoriasis.^248^ In January 2016, it received additional FDA approval for the treatment of active AS and PsA.^249^ Secukinumab, marketed by Novartis, was approved by the European Medicines Agency (EMA) in April 2020 to treat non-radiographic axial spondyloarthritis. Secukinumab is recommended for patients eligible for systemic therapy and/or phototherapy. The most common side effects of secukinumab are upper respiratory tract infections (8.1–8.8%), headache (10.4–11%), and nasopharyngitis (26.9–29.3%), followed by arthralgia, hypertension, diarrhea, back discomfort, pruritus, and cough.^250^

##### Brodalumab

Brodalumab inhibits all IL-17 cytokines (unlike secukinumab, which directly inhibits IL-17A production only) by preventing interactions with their receptors. Brodalumab (AMG 827) is a human IgG2 mAb that binds to IL-17 receptor A (IL-17RA), thereby inhibiting IL-17A, IL-17F, and IL17-A/F heterodimer signaling over the IL-17RA/RC complex and IL-17E signaling across the IL-17RA/RB complex.^251,252^ Inhibiting IL-17RA prevents IL-17-mediated release of proinflammatory chemokines and protein kinases.^253^ Brodalumab was approved for the treatment of moderate-to-severe plaque psoriasis by the Japanese Pharmaceuticals and Medical Devices Agency in July 2016 (Lumicef®), FDA in February 2017 (SiliqTM), and European Medicines Agency in July 2017 (Kyntheum®).^254^

##### Ixekizumab

Ixekizumab (Taltz®, LY2439821; Eli Lilley and Company) is a humanized IgG4 mAb that specifically binds IL-17A and prevents interactions with IL-17R. By targeting cells, it prevents the release of proinflammatory cytokines and chemokines, subsequently affecting cellular components. Ixekizumab does not interact with human Fc I, IIa, or IIIa receptors or the complement system’s building blocks. Ixekizumab received FDA and EMA approval in 2016^255^ for the treatment of moderate-to-severe plaque psoriasis, as well as active PsA (FDA in 2017, EMA in 2018) and AS (FDA in 2019).^256^

In addition, in vivo, the Th17/Treg balance is restored by inhibiting cytokines that inducing Th17 production or by promoting cytokines that induce Treg differentiation, thus indirectly affecting IL-17 production to achieve therapeutic benefits.^257,258^

Although IL-17 blockade is beneficial in some cases, the amount of antibody administered must be strictly controlled to avoid interference with the normal functions of IL-17 in the body and increasing the risk of opportunistic infections. In a trial using a secukinumab and brodalumab for treating Crohn’s disease, symptoms worsened in patients receiving anti-IL-17 antibodies, leading to early termination of the trial.^149,150,259^ As discussed above, IL-17 also plays an important role in establishing host barriers and maintaining mucosal integrity. The results of the above study supported the suggestion that IL-17 is essential in maintaining intestinal barrier integrity, which was disrupted by excessive blockade of the IL-17 signaling pathway.^153,260^ Second, although several different types of IL-17 inhibitors have been effective in mouse models, further studies are required to confirm their precise effects in humans. Finally, different immune diseases or even the same immune disease can have different mechanisms of action across individuals. Therefore, further research on the pathogenesis of various immune diseases and determining the cytokines that cause disease development may provide novel ideas for developing targeted drugs.

### IL-17 and neoplasms

In addition to autoimmunity, abnormal IL-17 levels are important causative factors in early- and late-stage human cancer development.^153,156^ Alterations in IL-17 levels have a significant impact on tumor development in a wide range of organs, including the colon,^261,262^ liver,^263^ pancreas,^264^ lungs,^265^ and bile ducts.^266^ Furthermore, preclinical and clinical studies have associated high serum IL-17 levels with poorer prognosis and radiotherapy resistance in patients with various solid tumors, suggesting that IL-17 inhibition may be effective in suppressing tumor metastasis and enhancing the sensitivity of cancer cells to radiotherapy.^267–270^ The same phenomenon is observed with chemotherapy and targeted therapies.^268,271–274^ However, some researchers believe that IL-17 can act as both a promoter and inhibitor of human tumor progression, and elucidating its inhibitory mechanism in cancers may also help to develop future tumor treatments.^275,276^

Current studies on IL-17 have focused on its essential role in tumor initiation, development, and regression.^277–279^ Recent evidence suggests that chronic inflammation may be a predisposing risk factor for many tumors.^280,281^ Chronic IL-17-induced inflammatory responses are considered as essential factors in mediating cellular carcinogenesis, promoting tumor cell proliferation and metastasis, and inducing immune tolerance in cancer cells. Abnormal cytokine expression has been detected in the tumor microenvironment, and IL-17 levels in these environments significantly differ from those in the surrounding normal tissues. IL-17 is overexpressed in both the tumor cells and tumor microenvironment of patients with malignancies.^267,268,282^ This differential IL-17 expression between tumorous and healthy tissues suggests that it has a biological role in the development of tumors.^283,284^ These findings suggest that IL-17-mediated signaling pathways may play essential roles in the transformation of a chronic inflammatory state into a malignant state.

#### Contribution of IL-17 to carcinogenesis

As previously mentioned, many studies have shown that tumor development is closely related to abnormal IL-17 expression. Specifically, IL-17 produced by chronic inflammation induces the formation of a tumor microenvironment, and promotes tumorigenesis and cancer progression by remodeling the cellular stromal phenotype and inducing the production of inflammatory mesenchymal stem cells and mobilizing myeloid cells.^285,286^ Chronic inflammatory stimulation caused by recalcitrant pathogenic microbes is associated with dysregulated IL-17 production in the microenvironment.^287^ This prompts continuous attempts by the host immune system to control an uncontrolled immune response. However, chronic inflammation is still triggered in cases where the underlying cause remains unclear. Such long-term and repeated bouts of inflammation increase the rate of genetic mutations and promote the development of precancerous cells, significantly increasing the probability of tumor development.^288,289^ Moreover, it has been suggested that tumors are essentially hard-to-heal wounds, and that repetitive tissue damage increases the incidence of IL-17-dependent malignancies.^290,291^ Whether long-term chronic inflammation or recurrent tissue damage is involved, the common factors are significant increases in cell proliferation and IL-17 levels. Therefore, there may be a potential link between IL-17 levels and cell proliferation. In this context, increased IL-17 signaling has been suggested to induce cancer stem cell (CSC) production by acting directly on myeloid stem cells to compensate for tissue regeneration and increase the proportion of precancerous cells, leading to tumor formation.^161,292^

**Fig. 6:**
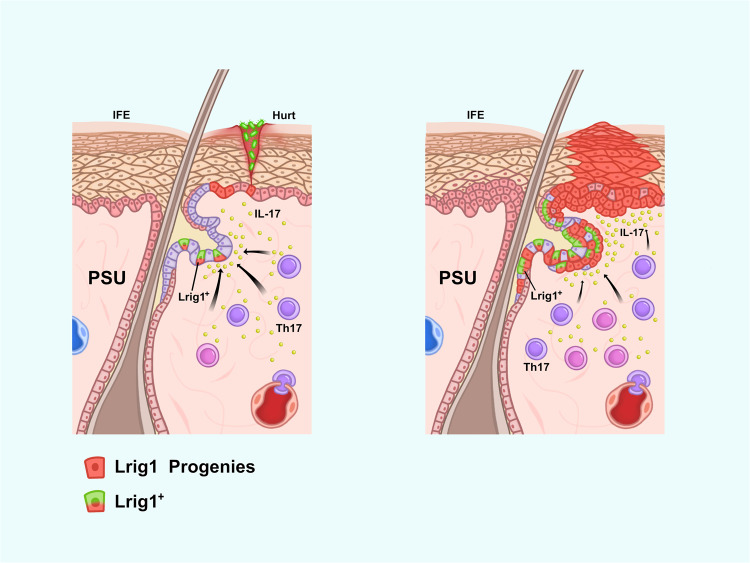
Injury and tumorigenesis. Damage to the interfollicular epidermis causes the body’s immune cells to migrate to the vicinity of the damaged tissue via the bloodstream and other routes. At this time, activated Th17 cells secrete large amounts of IL-17, which then act on Lrig1^+^ stem cells in the sebaceous gland, stimulating their activation and proliferation to repair the damaged interfollicular epidermis. However, Lrig1^+^ stem cells containing the oncogenic K-Ras G12D gene mutation form unique tumorigenic stem cells when activated by the IL-17 signaling pathway. In the presence of IL-17, Lrig1^+^ cancer stem cells proliferate and differentiate rapidly, forming tumors at the injury site. IFE, interfollicular epidermis; PSU, pilosebaceous unit; and SG, sebaceous gland. MediBang Paint was used to generate this figure

**Fig. 7:**
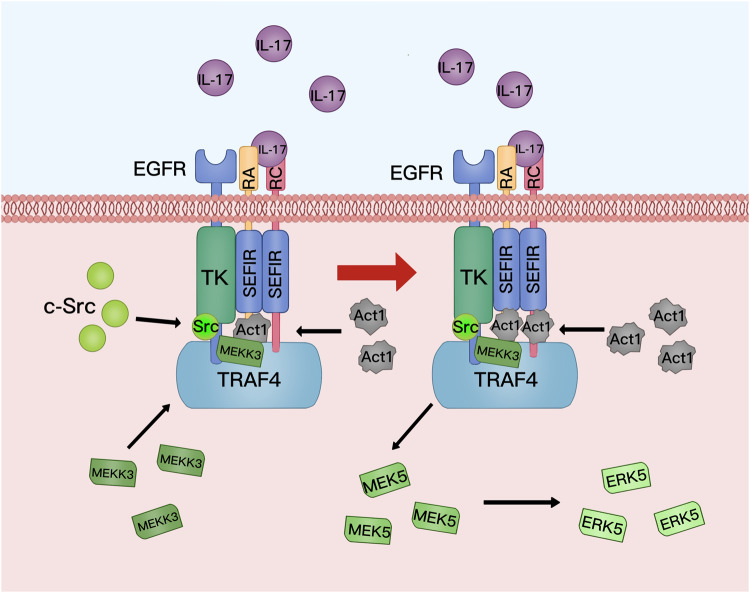
Activation of the IL-17R–EGFR axis. The IL-17R–EGFR axis begins with IL-17A binding to IL-17R (consisting of IL-17RA and IL-17RC). Ligand binding recruits EGFR for the IL-17A-mediated activation of Lrig1^+^ stem cells. TRAF4, which is enriched in Lrig1^+^ stem cells, binds tightly to the neighboring EGFR that is attached to the IL-17A–IL-17R complex. This tight binding allows Act1 to recruit c-Src for the IL-17A-induced EGFR transactivation and activation of the ERK5 downstream pathway. TK, tyrosine kinase domain; RA, IL-17RA; RC, IL-17RC; EGFR, epidermal growth factor receptor; TRAF, tumor necrosis factor receptor-associated factor; c-Src, a tyrosine kinase; MEK, mitogen/extracellular signal-regulated kinase; ERK, extracellular signal-regulated kinase; and MEKK, MAPK/ERK kinase kinase. MediBang Paint was used to generate this figure

The stem cell properties of tumor cells from head and neck squamous cell carcinoma are enhanced through epithelial–mesenchymal transition (EMT).^293^ However, whether the EMT pathway increases the stem cell potential of the body’s cells and thus induces tumorigenesis has not been confirmed. The most widely accepted hypothesis is that tumorigenesis is closely related to the appearance of CSCs. In mice, IL-17A-mediated signaling mobilizes leucine-rich repeats and immunoglobulin-like domains 1-expressing (Lrig1^+^) stem cells during wound healing and tumorigenesis.^161^ In normal murine skin, Lrig1^+^ stem cells are confined to the upper part (i.e., funiculus and sebaceous gland), which do not constitute the interfollicular epidermis of the pilosebaceous unit, where they are responsible for maintaining damage repair and tissue regeneration. When tissue injury occurs, IL-17A increases the number of Lrig1^+^ stem cells in the pilosebaceous unit by activating EGFR, promoting the migration of progenitor stem cells into the interfollicular epidermis for wound repair (Fig. 6). Mechanistically, IL-17A binding to its receptor (IL-17R) triggers the activation of EGFR, which subsequently binds to IL-17R. Tumor necrosis factor receptor-associated factor 4 (TRAF4), which is enriched in Lrig1^+^ stem cells, then binds to EGFR to form the IL-17R–EGFR–TRAF4 complex. Free molecules of the Act1 adapter protein bind to this complex to recruit the proto-oncoprotein c-Src for IL-17A-induced EGFR transactivation and activation of the downstream ERK5 signaling pathway (Fig. 7). In one study, when the oncogenic K-Ras G12D mutation was expressed in Lrig1^+^ cells, the injury site formed unique tumorigenic stem cells that rapidly transformed into tumors within a few days (Fig. 6).^173^ Given the critical function of IL-17A in Lrig1^+^ cell proliferation and tissue repair, studies have investigated the effects of knocking down the IL-17R-EGFR axis and TRAF4 expression. IL-17A-induced Lrig1^+^ cell expansion was reduced, and oncogenic K-Ras G12D-mediated activation of tumor formation was inhibited. This unique Act1-TRAF4-ERK5 signaling cascade, triggered by IL-17A, links wound healing to tumorigenesis via IL-17.^161^ In addition, IL-17-mediated signaling plays an essential role in regulating stem cell populations in other organs.^263,294–296^

#### Role of IL-17 in tumor progression

IL-17 also plays an essential role in tumor progression; the evidence suggests it promotes tumor cell proliferation in two ways. Firstly, it directly acts on tumor cells, where it regulates the expression of inflammatory factors, chemokines, and growth factors.^297^ IL-17 is a modest transcriptional activator in vitro. Its signaling pathway can maintain the stability of target genes through the Act1–TRAF2–TRAF5 complex or can promote tumor cell proliferation by regulating the Act1–TRAF4–ERK5 axis at the transcriptional and/or post-transcriptional levels.^17^ Secondly, IL-17 can act indirectly on tumor cells to regulate their proliferation, with the common mechanism being the promotion of blood vessel formation in the tumor microenvironment via induction of the secretion of pro-angiogenic factors and matrix metalloproteinases by tumor cells, stromal endothelial cells, and fibroblasts, thereby improving tumor cell access to nutrients.^265,298^ Furthermore, the role of host flora in tumors and autoimmune diseases has been investigated. The data suggests that tumors may share mechanistic routes with immune-mediated inflammatory diseases, such as multiple myeloma, by modulating the host microbiota and/or blocking IL-17.^299^

**Fig. 8:**
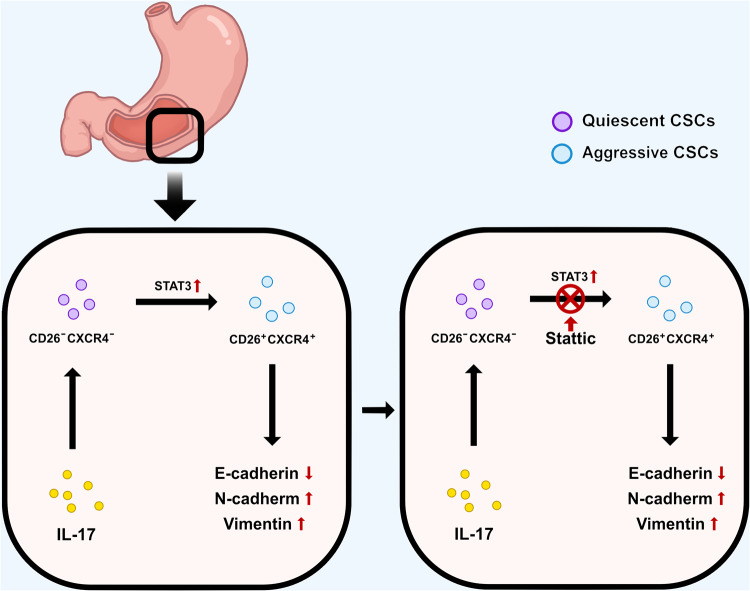
Transformation of quiescent gastric cancer stem cells (CSCs). Treatment of quiescent gastric CSCs (CD26^–^CXCR4^–^) with appropriate concentrations of exogenous IL-17 induces their conversion to aggressive CSCs (CD26^+^CXCR4^+^). At the same time, signal transducer and activator of transcription 3 (STAT3) expression are upregulated, and the expression of markers associated with the epithelial-mesenchymal transition (EMT) pathway is altered (i.e., E-cadherin expression is downregulated and N-cadherin and vimentin expression is upregulated). However, inhibition of the STAT3 signaling pathway via Stattic prevents the transformation of gastric CSCs from a quiescent to an invasive phenotype, and markers associated with the EMT pathway are not significantly altered. MediBang Paint was used to generate this figure

Local tumor invasion, recurrence, and distant metastasis are the leading causes of drug resistance and poorer prognosis.^300^ IL-17-mediated signaling is associated with the migration and invasion of various malignant tumors, including oral squamous cell carcinoma,^23^ breast cancer,^24^ and gastric cancer.^25^ In addition, EMT can effectively promote tumor metastasis, recurrence, and drug resistance by enhancing the stem cell properties and mobility of tumor cells. In recent years, IL-17-mediated CSC formation^161,301^ and EMT-like alterations have been suggested to be essential for tumor migration and invasion.^302^ Previous studies have suggested that gastric cancer originates from gastric tumor stem cells. One research group identified specific subpopulations of gastric CSCs with high metastatic capacity in clinical tissue samples of gastric cancers. The researchers classified them into quiescent (CD26^–^CXCR4^–^) and aggressive (CD26^+^CXCR4^+^) CSCs, and discovered that quiescent cells could transform into more aggressive cell types under specific circumstances.^303^ Another study revealed that CSCs with higher aggressiveness transformed from their quiescent counterparts through EMT-like changes, and that IL-17 likely induced this transformation via STAT3.^304^ In that study, researchers observed increased STAT3 expression and altered EMT marker (i.e., E-cadherin, N-cadherin, and vimentin) expression levels in IL-17-treated quiescent CSCs. Moreover, treatment with Stattic, a STAT3 signaling pathway inhibitor, downregulated the expression of EMT markers, revealing the importance of STAT3 in the EMT pathway. To further investigate the role of IL-17 in promoting EMT-like transformation in quiescent CSCs, the same researchers treated quiescent CSCs with appropriate concentrations of IL-17 and found that it increased cancer cell invasiveness, downregulated E-cadherin expression, and upregulated N-cadherin and vimentin expression, which contribute to EMT-like changes (Fig. 8). Moreover, tail vein injection of quiescent CSCs (treated with appropriate concentrations of IL-17) into nude mice resulted in a significant increase in lung metastasis. Western blot analysis revealed upregulated STAT3 expression and changes in EMT-related markers in these mice. In summary, both in vivo and in vitro experiments have shown that IL-17 promotes EMT-like changes in CSCs through the STAT3 pathway, which may be a contributory mechanism for tumor invasion and distant metastasis.^294^

By analyzing tumorigenesis, proliferation, and metastasis processes, we can identify a connection between IL-17 and CSCs that may have a crucial role in tumor progression. CSCs are rare normal tissues, even when damaged. However, the presence of IL-17 induces the conversion of healthy stem cells to CSCs, thus promoting tumorigenesis.^294,304^ Subsequently, IL-17 promotes the growth and reproduction of CSCs by acting on the tumor or its microenvironment, accelerating tumor progression.^305–308^ Moreover, IL-17 can act on quiescent CSCs to cause EMT-like changes and transform them into invasive CSCs, promoting tumor invasion and metastasis.^294^ Thus, IL-17 has an important role in tumor progression. We anticipate that ongoing and intensive research on IL-17 and CSCs will inform the discovery and development of new tumor treatments.

#### Contradictory antitumor role of IL-17

Although many published reports have confirmed the pro-tumor effects of IL-17s, some studies have suggested that the IL-17 family also has a unique antitumor role. Dorota et al.^309^ found that the balance between IL-17A and IL-17E can either facilitate or inhibit tumor progression. Other researchers found that the IL-17 family member IL-17D can inhibit tumor progression by recruiting natural killer cells to mediate tumor rejection.^310^ Moreover, lymphocytes can induce tumor cell death and proliferation by activating the p53 signaling pathway and secreting IL-17.^311^ Furthermore, although IL-17 promotes the formation of blood vessels in the tumor microenvironment to facilitate nutritional access by tumor cells for their growth and development, this vascular network also provides a direct pathway for antitumor immune cells to enter the tumor interior. These findings provide a biological basis for the antitumor effects of IL-17. We believe there is a correlation between the antitumor and pro-tumor effects of IL-17. First, the amount of IL-17 produced and its duration of action may determine its role in the body. When IL-17 acts for a short duration, it preferentially activates signaling pathways associated with the inflammatory response and may induce acute inflammation to remove pathogenic factors from the body. In contrast, at high levels and/or with longer durations of action, IL-17 activates inflammation-related signaling pathways and subsequently activates other signaling pathways, which in turn execute pro-tumorigenic functions. Moreover, because different body parts have different degrees of tolerance to IL-17, its levels and whether it is expressed at specific sites may play different roles in tumorigenesis and cancer development.

An illustrative example is the influence of IL-17 on lung cancer. IL-17, in contrast to the pro-tumorigenic features discussed above, can prevent tumor formation. The ability of IL-17 to recruit dendritic cells contributes to antitumor immunity in the B16 melanoma^312^ and Pten/Smad4 deficient lung cancer models.^313^ Furthermore, the molecular fingerprints of Pten/Smad4 deficient lung cancer cells^314^ differ from those of KrasG12D lung cancer cells,^315^ suggesting that IL-17 functions differently in these two tumor models.

Despite a thorough genomic and prognostic investigation of the IL-17 family genes in lung cancer, researchers did not elucidate the expression and function of IL-17A and IL-17B in lung cancer.^316^ This shows that IL-17 may have both pro- and anti-tumorigenic characteristics in the same type of cancer. IL-17D expression is considerably lower in lung cancer and is associated with improved survival. Research on the role of IL-17CF in lung cancer is scarce. More research on the relationship between IL-17D and lung cancer progression is required in order to identify effective lung cancer therapy targets.^316^

#### Roles of IL-17 in cancer diagnosis, immunotherapy, and drug resistance

Chemotherapy and radiotherapy, together with surgery, are still the major treatment options for many patients with advanced stage cancers.^317–319^ However, immunotherapy has shown promising efficacy against some tumors, and is gradually becoming a mainstream treatment option.^320–322^ IL-17 plays a role in promoting tumorigenesis, cancer progression, and metastasis through its unique signaling mechanisms. Thus, modulating IL-17 production and other IL-17-mediated signaling pathways will likely contribute to advancing tumor-specific immunotherapies and improving patients’ prognoses. Using a mouse model of K-RAS gene-induced lung cancer, Felix et al. found that IL-17C-mediated innate inflammatory responses enhance cancer cell resistance to anti-programmed cell death protein 1 (PD-1) immunotherapy, and that blocking the IL-17C signaling pathway may improve the outcomes of patients with lung cancer receiving PD-1 therapy.^323^ Similarly, Esra et al. showed that blocking IL-17 is an effective method for improving PD-1 therapy efficacy.^324^ Many clinical studies have indicated the role of IL-17 in tumors and the efficacy of anti-IL-17 therapy (Table 2). Furthermore, because IL-17 can be detected in the blood, bone marrow, and pleural fluid of patients with tumors, using IL-17 as a biomarker may be a minimally invasive method for the early screening and diagnosis of tumors. However, the current evidence regarding IL-17 as a diagnostic marker is insufficient, and clinical studies with large sample sizes are needed to verify its utility.

**Table 2:** Effect of IL-17 in tumor models and lines

Turmor type	Model and cell lines	Role of IL-17	Ref
Breast cancer	K14cre;Cdh1F/F;Trp53F/F PyMT/Tgfbr2KO MCF7/T47D/BT20/MDA-MB468/MDMB157/MDA-MB231	Pro-tumorigenic	^325–328^
	MCF7, MDA-MB468, MDA-MB 435-S, MDA-MB231, SKBR3, T47D, ZR75, Hs578t, HCC1937, MDA-MB175-7	Anti-tumorigenesis	^329,330^
	Cl66 cells resistant to doxorubicin (Cl66-Dox), or Cl66 cells resistant to paclitaxel (Cl66-Pac)	Resistant to doxorubicin and paclitaxel	^268^
Colorectal cancer	AOM DSS	Pro-tumorigenic	^14,82,331–333^
	APCmin	(IL-17F; anti-tumorigenic)	
	NOD/Shi-scid/IL-2Rγ null (NSG) mice *Cdx*2Cre-ERT2 *Apc*F/F mice	Pro-tumorigenic	
	Il17re-/- mice;Myd88-/-mice	Pro-tumorigenic	^334^
Hepatocelluar carcinoma	hURI-tetOFFhep (IL-17RA D/DMyeloid)	Pro-tumorigenic	^335^
	MHCC97L/HepG2 (cell lines)	Pro-tumorigenic (IL-17E)	^336^
Lung cancer	KrasG12D × CCSPcre (+ NThi challenge) (IL-17F; redundant)	Pro-tumorigenic	^337^
	PtenF/F SMAD4F/F×CCSPcre	Anti-tumorigenic	^313^
	IL-17C-/- mice TLR-2/4-/- mice	Pro-tumorigenic(IL-17C)	^338^
Pancreatic cancer	KrasG12D xMist1CreERT2± Cispain	Pro-tumorigenic	^264^
Prostate cancer	PtenF/F × prostate-specific probasincre	Pro-tumorigenic	^339^
Papilloma and squamous cell carcinoma	DMBA/TPA	Pro-tumorigenic	^156,340,341^
Multiple myeloma	Constitutive MYC expression in early B cells	Pro-tumorigenic	^342^
Head and neck carcinoma	Cal27/DDP and FaDu/DDP	Cisplatin-resistant	^343^
Lung cancer	KRAS gene-induced	Anti-PD-L1-resistant	^272^
Oral cancer	4NQO-mice	Anti-PD-1-resistant	^344^

In addition to the conventional therapeutic agents that act directly on the IL-17 signaling pathway, a different treatment perspective has attracted research attention. Because of the specific expression of IL-17R on the surface of some CSCs, some researchers have used IL-17 as a CSC marker for targeted therapies. Bie et al. combined IL-17 with tumor growth inhibitors for the long-term abrogation of IL-17RB-expressing CSC production, which strongly inhibited tumor growth in vivo.^325^ This finding validates the possibility of using IL-17RB as a target cell marker for cancer therapy, and provides a novel immunotherapy strategy for cancer treatment.

Drug resistance has made it difficult for many current drugs to treat recurrent tumors effectively. Therefore, the causes of drug resistance are being extensively studied. High IL-17 levels in tumor tissues have been associated with tumor drug resistance.^270,326^ As described above, IL-17 can act either directly on CSCs to promote tumor proliferation, metastasis, and recurrence, or indirectly by altering the composition of the tumor microenvironment, thereby modulating tumor cell growth and leading to their recurrence and drug resistance. Therefore, downregulating the expression of IL-17 or blocking its signaling pathway would effectively reduce the emergence of CSCs, inhibit tumorigenesis and tumor cell proliferation, decrease the occurrence of EMT-like changes in CSCs, and inhibit the metastasis and recurrence of tumor cells.

In summary, various studies have shown that IL-17 can act directly or indirectly to provide cancer cells with adequate nutrition, suitable growth environments, and relevant signals for tumorigenesis, tumor development, and metastasis, leading to disease progression. Through various mechanisms, IL-17 can support early tumor formation and metastasis, and drug resistance in mid- to late-stage tumors. Future detailed studies of the molecular mechanisms and biological functions of IL-17 in the tumor microenvironment will enhance our understanding of the biological processes of various types of cancer, and provide novel immunotherapy strategies for treating malignant diseases related to this important proinflammatory cytokine.

## Opinions and conclusion

IL-17 first drew attention as a cytokine that induces autoimmune diseases with characteristics of inflammatory disorders. Researchers then discovered its critical roles in the preservation of mucosal immunity and barrier integrity, which enabled not only the establishment of host defenses but also accelerate carcinogenesis and cancer progression under pathological conditions. These pathological roles are dependent on IL-17’s ability to produce proinflammatory mediators, act as a mitogen in tissue progenitor cells, and alter cellular metabolism. These consequences are influenced mechanistically at both the transcriptional and post-transcriptional stages.

For cancer research, managing advanced and metastatic cancers or cancers that recur after traditional therapy tools (i.e., surgery, chemotherapy, radiation, and tumor immunotherapy) are critical areas of interest. Unfortunately, the role of IL-17 in these cancers remains unclear. In addition, it is important to perform cell type-specific (i.e., tumor stem, endothelial, and other stromal cells) research on the role of IL-17 in the tumor microenvironment. The ontogeny and destiny of tumor-associated Th17 cells may differ greatly depending on the tumor type, as some cancers are known to be induced by tissue inflammation. Finding the best targeted medicines and therapy windows for cancer can be made easier by molecular examination of these pathways in the tumor environment.

Currently approved treatments targeting the IL-17-IL-17R pathway are all monoclonal antibodies. Some have been linked to negative side effects, such as depression and escalation of the immune system’s inflammatory response. Orally bioavailable small molecule drugs (SMDs) are beneficial in terms of production cost, convenience of delivery, and the potential for fewer infection-associated adverse effects. SMDs can transiently inhibit IL-17 production, whereas biologics, often, do so permanently. This may help to halt the cycle of inflammation without impairing IL-17’s ability to protect against infection. However, some SMDs can cause off-target toxicity. Because IL-17 plays both protective and destructive roles in inflammation; therefore, a more tailored strategy should be to employed to preserve the host’s own immunoregulatory systems.

In this paper, we review the latest findings on the IL-17 signaling pathway and comprehensively analyze and present its synergistic effect with other signaling molecules. We have summarized and discussed the different effects of IL-17 under physiological and pathological conditions. When the body is in a physiological state, IL-17 plays an important role in maintaining mucosal immunity and barrier integrity. IL-17 can maintain immune tolerance of the body, help establish host defenses, and promote tissue regeneration at damaged sites to maintain the integrity of the mucosal barrier, which is an essential factor for maintaining host health and homeostasis. However, under pathological conditions, the balance of IL-17 in the body is disrupted, and its secretion is increased. Excessive IL-17 production in the body leads to the occurrence and aggravation of some immune diseases. In addition, the IL-17-mediated signaling pathway is closely associated with the occurrence, proliferation, metastasis, and drug resistance of tumors under pathological conditions. Overall, IL-17 plays an important role in both physiological and pathological conditions, depending on its ability to induce proinflammatory mediators, tissue regeneration, and therapeutic resistance. This review aimed to provide researchers with a clear understanding of the role of IL-17. Further detailed and comprehensive research on IL-17-mediated signaling pathways under physiological and pathological conditions may provide prospects for the treatment of immune diseases and tumors.
